# Ultra-Stable Polycrystalline CsPbBr_3_ Perovskite–Polymer Composite Thin Disk for Light-Emitting Applications

**DOI:** 10.3390/nano10122382

**Published:** 2020-11-29

**Authors:** Saif M. H. Qaid, Hamid M. Ghaithan, Bandar Ali Al-Asbahi, Abdullah S. Aldwayyan

**Affiliations:** 1 Physics and Astronomy Department, College of Science, King Saud University, Riyadh 11451, Saudi Arabia; hghaithan@ksu.edu.sa (H.M.G.); balasbahi@ksu.edu.sa (B.A.A.-A.); dwayyan@ksu.edu.sa (A.S.A.); 2 Department of Physics, Faculty of Science, Ibb University, Ibb 70270, Yemen; 3 Department of Physics, Faculty of Science, Sana’a University, Sana’a 12544, Yemen; 4 King Abdullah Institute for Nanotechnology, King Saud University, Riyadh 11451, Saudi Arabia; 5 K.A. CARE Energy Research and Innovation Center at Riyadh, Riyadh 11451, Saudi Arabia

**Keywords:** amplified spontaneous emission, thin-disk, perovskite, stability

## Abstract

Organic–inorganic halide organometal perovskites have demonstrated very promising performance in optoelectronic applications, but their relatively poor chemical and colloidal stability hampers the further improvement of devices based on these materials. Perovskite material engineering is crucial for achieving high photoluminescence quantum yields (PLQYs) and long stability. Herein, these goals are attained by incorporating bulk-structure CsPbBr_3_, which prevents colloidal degradation, into polymethyl methacrylate (PMMA) polymer in thin-disk form. This technology can potentially realize future disk lasers with no optical and structural contributions from the polymer. The polycrystalline CsPbBr_3_ perovskite particles were simply obtained by using a mechanical processing technique. The CsPbBr_3_ was then incorporated into the PMMA polymer using a solution blending method. The polymer enhanced the PLQYs by removing the surface trap states and increasing the water resistance and stability under ambient conditions. In our experimental investigation, the CsPbBr_3_/PMMA composites were extraordinarily stable and remained strongly luminescent after water immersion for three months and air exposure for over one year, maintaining 80% of their initial photoluminescence intensity. The CsPbBr_3_/PMMA thin disk produced amplified spontaneous emission for a long time in air and for more than two weeks in water.

## 1. Introduction

Organic–inorganic (hybrid) and inorganic lead halide perovskite materials have significant potential in optoelectronic applications, including solar cells, photodetectors, and light-emitting devices. Inorganic cations (CsPbX_3_, where X is a halide) are incorporated as the active materials in perovskite, mainly in light-emitting diodes but sometimes in photodetectors and solar cells. In light-emitting applications, inorganic perovskites show more promise than organic perovskites. In particular, CsPbX_3_ provides a short exciton radiative lifetime, long carrier diffusion length, excellent charge transport properties, narrow emission linewidth, and high photoluminescence (PL) quantum yields (PLQYs), and is more thermally stable than organic perovskites such as methylammonium PbX_3_ or formamidinium PbX_3_ [1,2,3,4,5,6]. However, owing to their ionic nature, perovskite crystals are sensitive to moisture and air, and have a low melting temperature. This instability hinders their applications in general [7,8,9], and impedes the further development of CsPbX_3_ in optoelectronics applications. To improve the stability of perovskite in the ambient atmosphere, we must research and develop some protective strategies such as surface passivation and encapsulation of perovskite in organic polymer matrices or polymer blends, and in mesoporous inorganic dielectric materials [1,8,10,11,12,13,14,15,16,17,18,19,20,21,22,23,24,25,26,27]. The protective materials should be compatible with perovskite, and transparent to preserve the optical properties [28,29,30,31]. These requirements can be met by using PMMA polymers. Although bulk-structure perovskites offer some advantages, nanoscale perovskite provides additional advantages by virtue of its high crystallinity, itself conferred by reduced trap density and enhanced mechanical stability. Nevertheless, bulk perovskites as optical amplifiers remain promising, with some developmental growth.

Next-generation displays and lighting technologies require efficient optical sources that combine brightness, color purity, stability, and substrate flexibility.

In light-amplification applications, population inversion requires a very strong optical pumping regime of the active layers. Moreover, to reduce the cost of optically or electrically pumped devices, long excitation pulses (at least at the nanosecond scale) are required. Continuous-wave excitation, requiring thermally stable materials, is desired. A powerful strategy that improves the active-layer stability is thus demanded.

Material engineering of perovskites is crucial for achieving highly luminescent PL properties. The optical properties of a material depend on the crystal structure, composition, domain size, and surface chemistry of the material.

In this work, we investigate the feasibility of CsPbBr_3_ thin disks in light-emitting applications and as a gain medium. The CsPbBr_3_ was incorporated into the polymer PMMA by the solution blending method. Polycrystalline CsPbBr_3_ perovskite particles were simply synthesized using a mechanical processing technique. The resulting composition will help in developing a new class of thin-disk composites for ultrastable efficient light emission, without requiring nanoparticles. The structural and optical properties of the thin disks were determined by using various characterization techniques. The perovskite incorporation improved the active material of the thin disk, and hence the maintenance of their optical properties. The thin disk exhibited a low amplified spontaneous emission (ASE) threshold, and remained stable for more than one year in air. It was also strongly resistant to water. Therefore, CsPbBr_3_/PMMA thin disks are a potential route to thin-disk lasers.

## 2. Materials and Methods

### 2.1. Materials

Lead(II) bromide (PbBr_2_, 99.999% trace metals basis) and cesium bromide (CsBr, 99.999% trace metals basis) were purchased from Sigma Aldrich (Saint Louis, MO, USA). Polymethyl methacrylate (PMMA) was purchased from Sigma-Aldrich (Saint Louis, MO, USA) and was used as received. All materials were dissolved in toluene solution with a purity of 99.8% (Fluka, Buchs, Switzerland). All chemicals were used without further purification.

### 2.2. Synthesis of PMMA Polymer and CsPbBr_3_ Perovskite Thin Disk

Polycrystalline CsPbBr_3_ perovskite particles were obtained by a mechanical processing technique. In this method, a 1:1 molar ratio of the CsPbBr_3_ primary components—PbBr_2_ (367.01 mg; 1 mmol) and CsBr (212.81 mg; 1 mmol)—were mixed by grinding both components in a mortar for over one hour, yielding a polycrystalline material. The synthesis was conducted in air.

Next, the PMMA polymer (which is compatible with CsPbBr_3_) was diluted in a nonpolar solvent (toluene). After mixing the polymer in solvent with CsPbBr_3_ (10 mg/mL), the mixture was poured into a flat petri dish. The dish was covered with a cap (this step was mandatory) with a small opening for slow drying at room temperature. After a few days, the thin disk was dried and ready for use. When 1.8 g of PMMA polymer, 15 mL of solvent, and 1 mL of CsPbBr_3_ (10 mg/mL) were mixed and poured into a standard petri dish with an approximate diameter of 9 cm, the resulting film was 0.25–0.35 mm thick. The quantity of the mixture can be increased to form a 1 mm-thick disk.

### 2.3. Characterization of the Thin Disk

Structural Characterization: The crystal phases of the thin disk were characterized by using X-ray diffraction (XRD) on an X-ray diffraction system (Miniflex 600, Rigaku, Japan) with copper Kα radiation (*λ* = 1.5418 Å). The scanning angle (2*θ*) was ranged from 10° to 80° at a scanning rate (step size) of 0.02° at 3°/min^−1^.

Optical Characterization: For optical characterization, the absorption spectra of the samples were recorded by an ultraviolet–visible (UV–vis) spectrophotometer (V-670, JASCO, Tokyo, Japan), and the PL spectra were measured under a continuous wave (CW) 471-nm laser.

ASE Measurements: To investigate the ASE and lasing properties of the thin disk, the power-dependent ASE spectra were collected at the sample edges near the ends of the excitation strips. For this propose, the pumping laser beam was focused to a radius of ~2 mm through a reflective microscope objective lens with a focal length of 10 cm, to reduce the divergence loss of the laser signal. The excitation beam from the laser unit has a beam diameter of ~1 cm. The sample was placed on a 6-degree-of-freedom adjustable stage.

Next, the laser beam was focused on the sample surface by using a cylindrical lens to form a rectangular stripe. The emission is collected at an angle of 90 degrees with respect to the optical axis set by the excitation laser beam. This geometry of the measurement maximizes the efficiency of the emission collection from the sample and reduces the background due to the excitation light. The amplification degree in the edge emission was much larger than that in the surface emission due to the much longer optical pass in the edge emission configuration, even though their optical gains were identical. Then, the signals emitted by the samples were directed through an optical fiber optic-coupled compact unit (Ocean Optics QE65 Pro, Dunedin, FL, USA). The energy density of the pumping laser was adjusted using a variable neutral-density filter wheel. The energy of the laser was measured using an LM-P-209 coherent thermal sensor head. By attenuating the laser energy density, we could study the effect of laser energy on the detection threshold.

Excitation was elicited by the third harmonic generation of a LOTUS II Q-switched Nd: YAG picosecond laser (LOTIS, Minsk, Belarus), with a pulse duration of 70–80 ps at a repetition rate of 15 Hz. The signals emitted by the samples were directed through an optical fiber and a collimating lens. In the lasing and ASE measurements, the detector was a QE65 Pro spectrograph (Ocean Optics QE65 Pro, Dunedin, FL, USA) with a spectral resolution of 0.78 nm. All measurements were performed in air at room temperature (293 K).

Time-Resolved PL Measurements: The time-resolved PL measurements were performed under excitation by the third harmonic (355 nm) of a pulsed laser. Special filters were used to cut the laser excitation pulse from the detector and to select the wavelength emitted from the sample. The emission light was collected through a lens and focused on a sufficiently sensitive fast photodiode (APD110). The time-scale decays were measured by a fast Tektronix TDS 380 two-channel digital real-time oscilloscope (bandwidth 400 MHz, sample rate 2 GS/s) operating in singles hot mode, which also provided the data output. The time base was 1ns to 5s/div with ±0.01% accuracy and 2000 sample/second.

Data Analysis: The large amount of data collected in the study were analyzed by a customized Python-based program developed by our research group. This program rapidly provided the Gaussian fits of dual PL emission peaks in multiple data files.

## 3. Results

### 3.1. Structural Characterization

**Figure 1:**
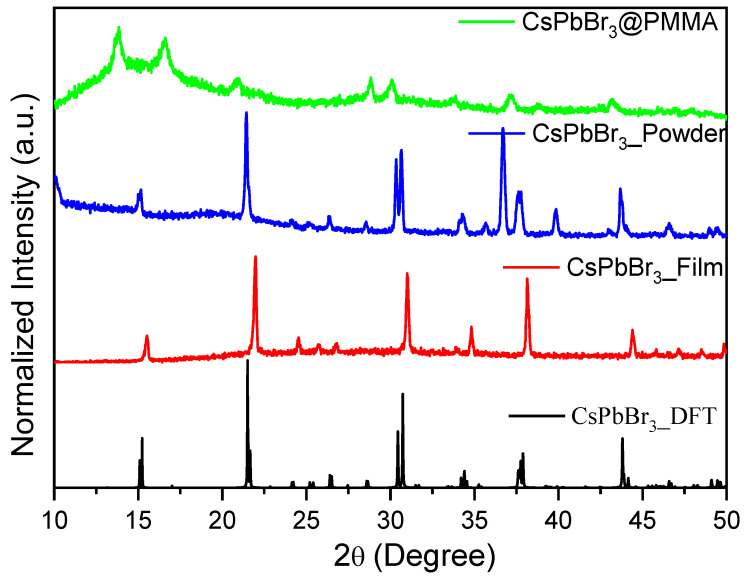
X-ray diffraction (XRD) patterns of a CsPbBr_3_ perovskite thin disk, as-ground CsPbBr_3_, thin film prepared in a thermal evaporation system, and density functional theory (DFT)-simulated CsPbBr_3._

The CsPbBr_3_ perovskite was prepared by a mechanical route that yielded perovskite particles in powder form. The X-ray diffraction (XRD) spectra of the synthesized perovskite particles are presented in Figure 1. This figure compares the X-ray diffraction patterns of CsPbBr_3_ simulated by density functional theory (DFT), the CsPbBr_3_ thin film formed in the thermal evaporation system, grounded CsPbBr_3_ powder, and the CsPbBr_3_/PMMA thin disk. The simulated XRD patterns were calculated in VESTA software using the lattice parameters with a Pnma space group, which describes the orthorhombic structure of CsPbBr_3_ perovskite at room temperature with a size of several hundred nanometers [32,33,34,35,36,37,38,39,40,41]. The XRD peaks of the perovskite particles prepared by the mechanical route closely matched those of CsPbBr_3_ perovskite thin-film material formed by a different synthesis method (namely, by thermal evaporation).

In the XRD patterns of the sample prepared by the mechanical route of perovskite and the CsPbBr_3_/PMMA thin disk, the peaks at 16.62°, 20.92°, and 43.14° ideally matched those of the simulated XRD pattern (Figure 1), which identifies the CsPbBr_3_ phase of perovskite as the majority constituent with no evidence to formed the Cs_4_PbBr_6_ phase [42,43,44], which is in very good agreement with the values reported in the literature [42,43,44]. The dominant phase of 3D CsPbBr_3_ perovskite can be confirmed by the present of the pervious XRD patterns. The absence of a peak at 2*θ* = 12.80° gives additional evidence for that the 0D phase (Cs_4_PbBr_6_) was not formed. The slight differences in the relative intensities of the XRD peaks of the perovskite materials made by different procedures can be explained by changes in the crystal orientations of the samples. No detectable amounts of the starting CsBr and PbBr_2_ materials were observed. Also, minor displacements in the peak positions can be attributed to the formation of additional crystal structures, indicating the coexistence of two closely spaced lattice planes, rather than to the formation of additional phases. This confirmed that the single crystalline phase was formed. The peaks can also be displaced by the high quantity of non-crystalline PMMA in the composite (CsPbBr_3_/PMMA = 0.56 wt.%). Interestingly, the XRD peaks clearly differed in their sharpness. These differences are associated with the particle sizes, which were deduced from the full width at half maximum (FWHM) of the peaks using the Scherrer equation. The calculated CsPbBr_3_ particle sizes are listed in Table 1. More grain-sized particles appeared in the CsPbBr_3_/PMMA state than in the powder form. Despite this, these findings reveal that PMMA has a very minor effect on the crystallization of CsPbBr_3_ which is attributed to the fact that the grain size in both states was comparable and in the range of experimental errors.

As demonstrated by the powder X-ray diffraction measurements, the mechanical synthesis strategy yielded high-quality crystalline CsPbBr_3_ material.

### 3.2. Optical Characterization

**Figure 2:**
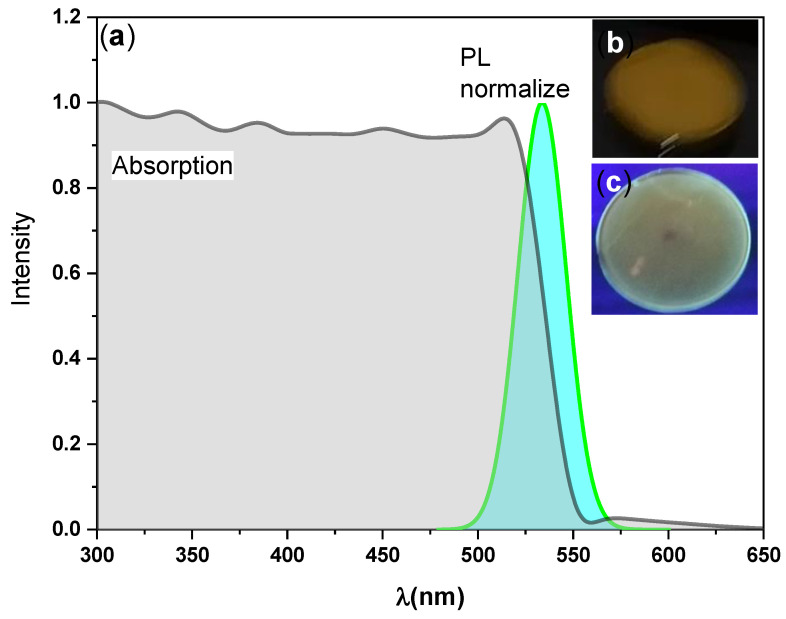
(**a**) UV-Visible absorption spectra and steady-state photoluminescence (PL) spectra of the CsPbBr_3_/PMMA thin disk. Insets (**b**) and (**c**) are photographs of the prepared thin-disk taken under natural and UV light, respectively, observed through an optical microscope.

The optical properties of the synthesized perovskite CsPbBr_3_/PMMA thin disk were characterized by steady-state absorption and PL spectroscopy. The optical absorbance spectra of the thin disk were recorded in the 300–650 nm region of the electromagnetic spectrum (see Figure 2). The absorption curve exhibited three broad features: A sub-bandgap absorption tail at low energy, followed by a strong exciton peak, followed by band-to-band transitions [45,46]. A broad absorption band with an exciton peak position at 2.30 eV was observed, and analysis of the UV-Visible absorption spectra confirmed a direct band gap of 2.30 eV (as discussed later). This finding also confirmed the presence of the 3D CsPbBr_3_ perovskite with dominant single crystalline phase, due to the absence peak at 324 nm, which related to the band–band excitation of 0D Cs_4_PbBr_6_ crystal [42,47]. This observation is in a good agreement with the pervious XRD results. Green fluorescence was observed by the naked eye (see the photographs of the thin disk taken under natural and UV lights in Figure 2, insets).

**Figure 3:**
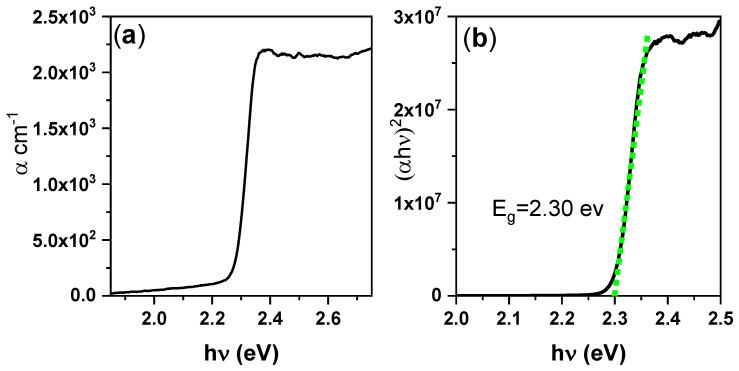
(**a**) Absorption coefficient spectra and (**b**) PL spectra of the CsPbBr_3_/PMMA thin disk.

The absorption coefficient α can be investigated through the Tauc relation. The direct and indirect transitions in the band gap of the material are then obtained by analyzing the optical absorption spectrum and applying the following formula [48]:

*αhν* = *C*(*hν −E*_g_)*^n^* with *α* = 2.303*A*/*d*, (1)

where *A* and *d* are the absorbance and thickness of the film, respectively, *h* is Planck’s constant, *ν* is the incident radiation frequency, *C* is a constant that depends on the transition probability, *E*_g_ is the energy band gap, and *n* = 1/2 and 2 for direct and indirect band gaps, respectively. The *α* spectrum thus derived from the absorption spectra is shown in Figure 3a. The low value of *α* (10^3^ cm^–1^) is attributable to the disk thickness. Assuming a direct bandgap, the band gap energy *E*_g_ of the perovskite thin disk was estimated from the intercept of the obtained straight lines at zero absorption, as shown in Figure 3b. The absorption of the sample corresponded to a direct energy band gap of 2.30 eV. The absorption and PL parameters are summarized in Table 2.

#### Steady-State and Time-Resolved Photoluminescence (PL) Analysis

**Figure 4:**
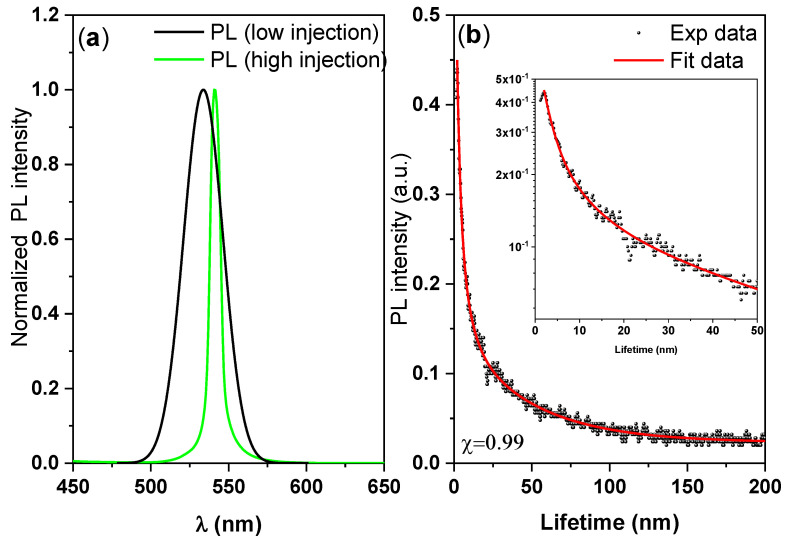
PL measurements: (**a**) Steady-state PL spectra and (**b**) time-resolved PL of the CsPbBr_3_/PMMA thin disk.

Figure 4 displays the steady-state PL spectra (black curve), the ASE spectrum (green curve), and the time-resolved PL of the CsPbBr_3_ thin disk. From these data, we can investigate the radiative recombination behavior, PL dynamics, and the defect healing effect in the sample. Figure 4a shows the normalized PL spectra in the low and high injection cases. The FWHM of the emissions decreased from 16.37 nm at low injection (spontaneous emission) to 7.88 nm at high injection (stimulated emission). The PL peak of the CsPbBr_3_/polymer thin disk (at 534 nm) was slightly redshifted from that of pure CsPbBr_3_ (at 520 nm). The redshift can be attributed to the aggregation of perovskite particles, which facilitates non-radiative recombination.

The difference between the peak energy of the PL before ASE threshold (spontaneous emission) and the ASE peak energy at the ASE threshold was 30 meV, indicating a high binding energy. Such an energy shift of the ASE relative to the spontaneous emission is usually considered to indicate the type of the transition mechanism from spontaneous emission to ASE. In our recent report [49], we referred to the fact that there are many factors that could have been responsible for the ASE red shifts, such as a re-absorbance effect, thermal effects, defect transitions, and bandgap renormalization.

The time-resolved PL studies were conducted under a low-fluence laser excitation pulse to avoid saturation effects. Representative time-resolved PL data are shown in Figure 4b. Each PL lifetime τ_i_ and its fractional amplitudes a_i_ were analyzed using the tri-exponential model. The PL decay profile was fitted at the peak position to the tri-exponential decay formula I (t) = a_1_ exp(−t/τ_1_) + a_2_ exp(−t/τ_2_) + a_3_ exp(−t/τ_3_), and the average lifetime was calculated as τ_avg_ = ∑a_i_τ_i_^2^/∑a_i_τ_i_. By utilizing fractional intensities (a_i_τ_i_), this method diminishes the influence of the fastest decay dynamics and increases the reliably of analysis. In the decay formula, τ_1_ and τ_2_ are the short-lived lifetime attributed to trap-assisted and contributed by surface-state non-radiative recombination, τ_3_ is the long-lived lifetime radiative recombination, and a_1,_ a_2,_ and a_3_ are the fractions of the three contributions [50,51,52]. The multiple lifetime components correspond to different conformations. The model fitting parameters are listed in Table 3.

The fast-decay components (short-lived lifetime) *τ*_1_, *a*_1_ and *τ*_2_, *a*_2_ are related to the trap-assisted non-radiative recombination at the grain boundaries, whereas the slow-decay components (long-lived lifetime) *τ*_3_ and *a*_3_ are related to radiative recombination inside the grains [50,51,52]. The fastest decay has very large fractional amplitude, and its dynamics provide little contribution to the total PL intensity, but it does markedly influence the fractional amplitudes of longer lifetimes, which is in a good agreement with literature [52]. The time-resolved single-photon counting measurements revealed that 58% of the charges recombined with a lifetime of 2.26 ns. The average PL decay time was around 35 ns. These data clearly indicate that under high injection conditions, increases in the steady-state PL signal do not necessarily correlate with decreased surface recombination rates.

### 3.3. Amplified Spontaneous Emission (ASE)

**Figure 5:**
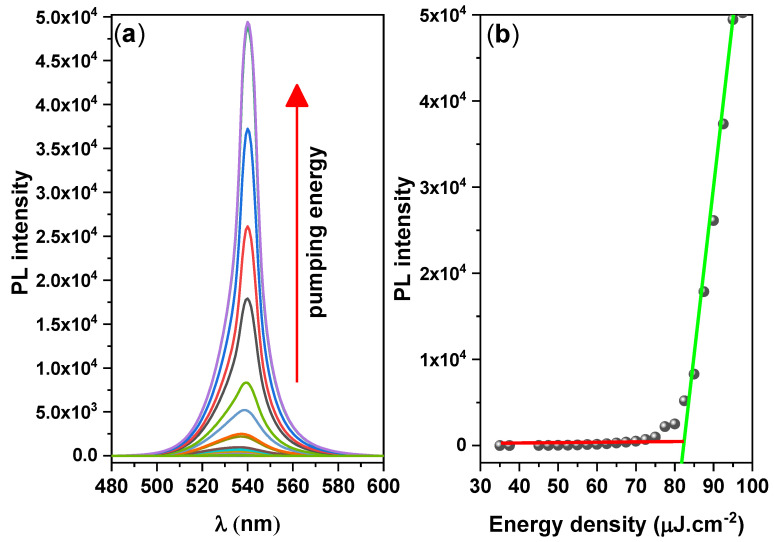
(**a**) Pump–fluence relationship and (**b**) Integrated PL intensity vs. pulse energy density, for the CsPbBr_3_/PMMA thin disk.

By analyzing the ASEs properties, we could examine the suitability of the CsPbBr_3_/PMMA thin disk as a gain medium when pumped to produce a population inversion. A cavity-free configuration allows the identification of behavioral variations induced by the material, rather than by the resonant cavity. To determine the ASE properties of the CsPbBr_3_ thin disk, we increased the pump fluence of the ASE measurements (Figure 5). At low pump energy, the PL spectrum was broad and featureless, but when the pump energy exceeded a critical point called the ASE threshold, a sharp and narrow peak appeared, indicating the occurrence of the transition from spontaneous emission to stimulated emission (ASE state), with the absence of standing longitudinal modes assessed using a laser resonator. The energy density threshold of ASE pumping was determined by the simultaneous occurrence of three phenomena: spectral narrowing of the emission, a nonlinear increase in emission intensity versus pump energy density, and an ASE peak near the long-wavelength region of the spontaneous emission, which grew very rapidly. The ASE parameters are listed in Table 4.

**Figure 6:**
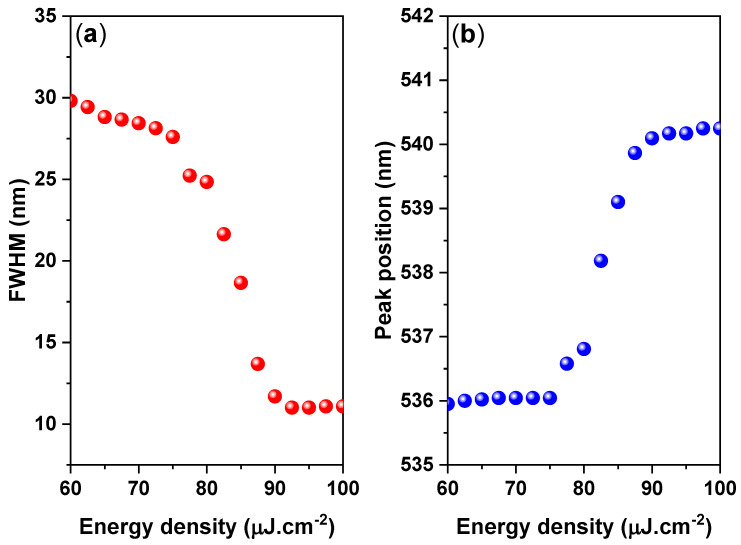
(**a**) FWHM and (**b**) ASE peak position versus energy density plot of the CsPbBr_3_/PMMA thin disk.

The onset of stimulated emission in the CsPbBr_3_/PMMA thin disk manifested as an immediate increase in the ASE intensity and a narrowing of the emission spectrum (FWHM ≈ 10 nm) over the threshold pumping range. The ASE generated at 537 nm shifted to 541 nm. The ASE peak (4 nm) was redshifted from the PL peak, as shown in Figure 6. However, when the pump energy increases above the ASE threshold, a redshifted peak has multiple causes, such as thermal effects, defect transitions [53], and band gap renormalization in the highly excited perovskite crystal [54], whereby the band gap is redshifted by hole–electron interactions under high population conditions. Moreover, as suggested by the re-absorption effect arising from the overlap of the absorption band edge with the PL emission (spontaneous emission spectrum) (Figure 2), the self-absorption effect should contribute to the ASE state [55].

#### Stability Studies

**Figure 7:**
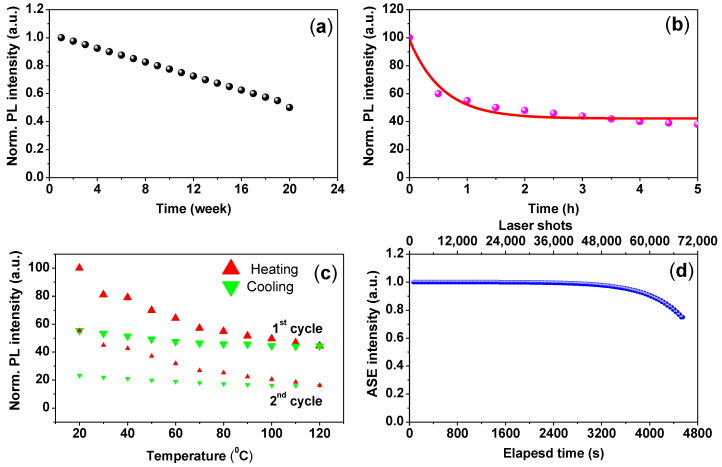
(**a**) PL intensity versus immersion time in water; (**b**) time-dependent PL intensity under UV lamp excitation; (**c**) temperature-dependent PL intensity of the composite in both stage heated and cooled; (**d**) shot-dependent ASE intensity of the CsPbBr_3_/PMMA thin disk.

To examine the water resistance and UV degradation of the CsPbBr_3_/PMMA thin disk, the disk was immersed in a water bath and exposed to UV-lamp excitation for five hours. Figure 7a plots the PL intensity as a function of immersion time. The PL intensities dropped to 50% after 4 months. Meanwhile, Figure 7b displays the time-dependent PL intensity of the CsPbBr_3_/PMMA thin disk under UV excitation for five hours in air. The PL of the perovskites degraded under the UV illumination due to reactions with air, causing photo-oxidation.

The thermal stability, another crucial index of the CsPbBr_3_/PMMA composite, was examined by monitoring the PL spectra under thermal effects. The experimental was carried out in Liquid Helium Cooled Cryostat (~10–300 K). To determine the thermal response of the sample, we plotted the temperature-dependent PL intensity of the composite during heating and cooling (Figure 7c). In this analysis, the composite disk was heated to 120 °C, and then cooled to room temperature (20 °C). The thermal stability was reflected in the PL spectra. The PL intensity of the composite decreased and increased as the temperature was raised and lowered, respectively. After cooling from 120 °C to 20 °C, the PL intensity was less than 40% of its initial value. Through repetition of the thermal cycle, the PL intensity exhibited a similar trend and the PL peak became stronger with increasing the cycles. By noting that the PL lost 30% of its original intensity compared to 40% in the first cycle. This decrease in the PL intensity is expected due to the reduction in the weight ratio of CsPbBr_3_/PMMA (<1 wt.%) and for heating over of the T_g_. The polymer-dependent thermal stability is correlated with the glass transition temperature *T*_g_ of the polymer (*T*_g_ = 105 °C for PMMA polymer) [50]. The *T*_g_ is an important determiner of structural integrity and geometry. Below the *T*_g_ of the polymer, the composite is nearly rigid because the polymer chains are frozen, whereas at the T_g_, the polymer was deformed. This phenomenon explains the low decay rate of the PL intensity upon heating and the high reversibility below 120 °C. The *T*_g_ of the CsPbBr_3_/PMMA composite was determined at 115 °C, where the plastic deformation was started. The *T*_g_ of the CsPbBr_3_/PMMA composite was lower than in previous reports [50] because the weight ratio (0.56 wt.%) differed in our study.

Finally, the CsPbBr_3_/PMMA thin disk achieved continuous ASE operational stability against photo-degradation and high optical gain characteristics. In general, photo-stability is evaluated by recording the time course of the total ASE intensity emitted under a constant pump intensity just above the threshold. Photo-degradation is detected as a decrease in the total ASE output. Figure 7d shows the ASE results of the thin disk. After 68,700 shots, the ASE intensity of the CsPbBr_3_/PMMA thin disk decreased to 73% of its initial value. Therefore, we can expect a long ASE lifetime (1/*e*) when the CsPbBr_3_/PMMA composite operates in air, which demonstrates the remarkable photo-stability of the composite. 

## 4. Conclusions

We investigated the feasibility of the CsPbBr_3_ thin disk in light-emitting applications and as a gain medium. The polycrystalline CsPbBr_3_ perovskite particles were obtained by a simple mechanical processing technique. The CsPbBr_3_ was then incorporated into the polymer PMMA using a solution blending method, and the composite was formed into a thin disk. The structural and optical properties of the CsPbBr_3_/PMMA composite were investigated by different characterization techniques. Next, the role of CsPbBr_3_ perovskite incorporated into PMMA was investigated by examining the ASE properties and optical response of bulk-phase perovskite CsPbBr_3_. Judging from the ASE of the CsPbBr_3_ disk, efficient light emission can be achieved not only by using CsPbBr_3_ perovskite nanoparticles, but also by using bulk CsPbBr_3_ perovskite. We found that incorporating perovskite into the polymer improves the properties of the active material; consequently, the optical properties of the CsPbBr_3_/PMMA thin disk were well maintained. The ASE threshold was low and the ASE output remained stable for over one year in air. Furthermore, the thin disk was water resistant. The resulting composition promises a new class of thin-disk composites (alternative to nanoparticles) with ultrastable efficient light emission. This work demonstrates a potential approach for fabricating thin-disk lasers.

## Figures and Tables

**Table 1:** Sizes of the CsPbBr_3_ thin disk, calculated from the XRD patterns using the Scherrer equation.

Sample	FWHM (Degrees)	Diameter (nm)
CsPbBr_3_-powder	0.314	33.00
CsPbBr_3_@PMMA	0.417	57.00

**Table 2:** Absorption and PL parameters of the CsPbBr_3_/PMMA thin disk.

Bandgap Energy *E*_g_ (eV)	PL Peak (eV)	Stokes Shift (meV)	FWHM of PL Peak (meV)
2.30	2.32	45.03	131

**Table 3:** Summarized PL lifetimes and their fractions in the CsPbBr_3_ thin disk, calculated by the tri-exponential decay model.

$\tau_{1}\;\left(ns\right)$	$a_{1}\;\left(\%\right)$	$\tau_{2}\;\left(ns\right)$	$a_{2}\;\left(\%\right)$	$\tau_{3}\;\left(ns\right)$	$a_{3}\;\left(\%\right)$	$\tau_{avg}\;\left(ns\right)$
2.26	57.75	7.12	23.94	46.02	18.31	35.23

**Table 4:** ASE parameters of the CsPbBr_3_/PMMA thin disk.

ASE Peak (nm)	FWHM of ASE_th_ Peak (nm)	Threshold (µJ cm^−2^)
537–541	10	82
